# κ-opioid receptor: from analgesic target to neuroimmune hub

**DOI:** 10.3389/fphar.2025.1744231

**Published:** 2026-01-07

**Authors:** Qinsi Tan, Mengjin Zheng, Huaixin Xing

**Affiliations:** 1 Graduate School of Shandong First Medical University and Shandong Academy of Medical Sciences, Jinan, Shandong, China; 2 Department of Anesthesiology, Shandong Cancer Hospital and Institute, Shandong First Medical University and Shandong Academy of Medical Sciences, Jinan, Shandong, China

**Keywords:** biasedagonism, immune cells, neuroimmunomodulation, signal transduction, therapeutic target, κ-opioid receptor (KOR)

## Abstract

Originally characterized as a classical mediator of analgesia, the κ-opioid receptor (KOR) has recently emerged as a pivotal regulator at the crossroads of the nervous and immune systems. Beyond its canonical role in nociceptive processing, a growing body of evidence reveals that KOR exerts profound immunomodulatory effects. The receptor is broadly expressed across diverse immune cell populations, including macrophages, microglia, and lymphocytes, where it contributes to immune homeostasis by attenuating the activity of key pro-inflammatory transcription factors, notably nuclear factor κB (NF-κB) and signal transducer and activator of transcription 3 (STAT3). These regulatory effects are mediated through both canonical G protein–coupled (Gαi/o) pathways and non-canonical β-arrestin–dependent cascades. Preclinical investigations have demonstrated that pharmacological modulation of KOR confers significant therapeutic benefits in a range of immune-related disorders, including atopic dermatitis, multiple sclerosis, and osteoarthritis. However, the clinical translation of traditional KOR agonists remains limited by dose-dependent central nervous system (CNS) adverse effects, such as dysphoria and hallucinations. In this review, we synthesize recent advances in elucidating the molecular and cellular mechanisms underlying KOR-mediated immunoregulation, highlight its therapeutic potential across diverse neuroimmune pathologies, and discuss innovative pharmacological strategies, such as peripherally restricted and signaling-biased ligands-designed to preserve beneficial immunomodulatory and analgesic properties while minimizing CNS liabilities. Collectively, these insights redefine KOR as a central node in neuroimmune communication and point toward the development of next-generation precision therapeutics targeting this axis.

## 1 Introduction

Cross-talk between the nervous and immune systems has emerged as a central paradigm in modern biomedical research. Among the molecular interfaces that mediate this bidirectional communication, the opioid system—defined by the widespread expression of its receptors and endogenous peptide ligands within both neural and immune compartments—serves as a prototypical example of neuro-immune integration. Within this system, the κ-opioid receptor (KOR) has attracted increasing attention as a crucial mediator linking nociceptive signaling with immune regulation.

Historically, KOR was originally characterized as a distinct receptor subtype primarily responsible for mediating analgesia (Martin et al., 1976). Abundantly expressed at several levels of pain circuitry (Simonin et al., 1995), KOR activation inhibits voltage-gated calcium channels and activates G protein-coupled inwardly rectifying potassium channels (Al- et al., 2011). This leads to neuronal hyperpolarization and the suppression of excitatory neurotransmitter release, effectively dampening pain transmission, particularly in models of visceral and inflammatory pain (Rivière, 2004). However, despite robust antinociceptive efficacy in rodents (Vonvoigtlander et al., 1983), KOR agonists have largely failed as mainstream analgesics in human clinical trials. The clinical utility of prototype agonists, such as spiradoline and enadoline, was severely compromised by a narrow therapeutic index, where dose-limiting central adverse effects—including dysphoria, sedation, and psychotomimetic reactions—emerged at or below analgesic doses (Pande et al., 1996). This translational failure has catalyzed a paradigm shift, compelling the field to look beyond central analgesia toward peripheral and neuroimmune mechanisms.

While traditionally studied within the central nervous system (CNS), KOR is now recognized to exhibit extensive peripheral expression, including within the gastrointestinal tract, the peripheral nervous system, and a diverse range of immune cell subsets (Szczepaniak et al., 2022). Functional expression of KOR has been identified in macrophages, microglia, T and B lymphocytes, dendritic cells, and neutrophils (Suzuki et al., 2001). This broad distribution underpins its capacity to modulate both neuronal excitability and immune activation, positioning KOR as a compelling therapeutic target in disorders that bridge neurological and immunological dysfunction—such as atopic dermatitis (AD), multiple sclerosis (MS), and other neuro-inflammatory conditions.

Compared with μ-opioid receptor (MOR) agonists, KOR-selective agonists confer distinct pharmacological advantages, including markedly reduced abuse potential and fewer respiratory or gastrointestinal side effects (Che and Roth, 2021). Consistent with these properties, preclinical and early clinical studies have demonstrated that KOR modulation exerts therapeutic benefits across a spectrum of conditions encompassing chronic pain, pruritus, neuroinflammation, and autoimmune diseases (Sun et al., 2019; Dalefield et al., 2022). Nevertheless, the broader clinical translation of KOR-targeted therapies remains hampered by characteristic CNS-mediated adverse reactions, most notably dysphoria and hallucinations (Sun et al., 2019; Dalefield et al., 2022). This paradox raises a fundamental challenge: how can the potent analgesic and anti-inflammatory effects of KOR activation be preserved while mitigating its undesirable central outcomes?

Addressing this question, the present review advances the conceptualization of KOR as a neuroimmune hub-a dynamic regulatory node coordinating the reciprocal interactions between the nervous and immune systems. We first delineate the molecular signaling mechanisms underlying KOR-mediated immunomodulation and then examine its cell type-specific actions across the innate and adaptive immune landscape. We further synthesize evidence supporting the therapeutic utility of KOR modulation in diverse pathological contexts, including neuroimmune skin disorders, central neuroinflammatory diseases, and malignancies. Finally, we discuss emerging pharmacological strategies-such as the design of peripherally restricted and signaling-biased agonists-aimed at decoupling therapeutic efficacy from central side effects, thereby paving the way for next-generation neuroimmune–targeted therapeutics.

## 2 KOR signal transduction: the molecular basis of immunomodulatory function

**FIGURE 1:**
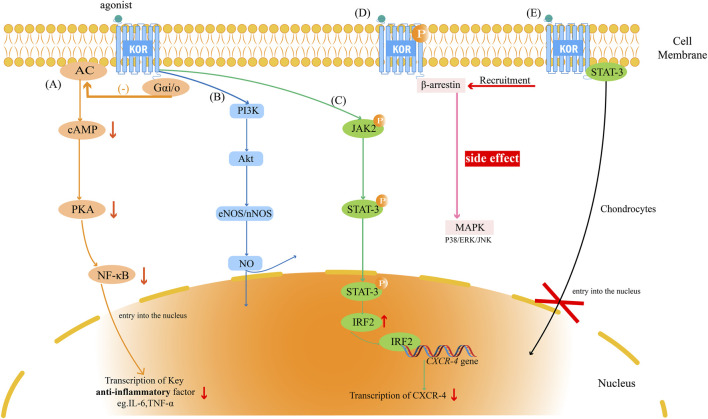
The KOR signaling network and its key biological effects on immune cells. κ-opioid receptor (KOR) activation initiates multiple parallel signaling pathways. **(A)** The inhibitory G protein/cyclic adenosine monophosphate (Gαi/o-cAMP) Pathway: This pathway is the canonical inhibitory pathway responsible for mediating anti-inflammatory effects by inhibiting the adenylate cyclase (AC)/cAMP/protein kinase A (PKA) axis, ultimately resulting in the inhibition of nuclear factor κB(NF-κB) activity. **(B)** The Phosphoinositide 3-kinase/Protein Kinase B (PI3K/Akt) Pathway: KOR activates the PI3K/Akt pathway, promoting cell survival and activating downstream endothelial Nitric Oxide Synthase (NOS)/nNOS to produce nitric oxide (NO). This pathway plays a key role in tissue protection. **(C)** The Janus kinase 2(JAK2)/Signal Transducer and Activator of Transcription 3(STAT3) Pathway: KOR activates JAK2, which in turn phosphorylates STAT3. Activated STAT3 upregulates Interferon Regulatory Factor 2 (IRF2) transcription upon nuclear translocation. The IRF2 protein acts as a transcriptional repressor, binding to the promoter of the C-X-C chemokine receptor type 4 (CXCR4) gene and downregulating its expression. **(D)** The β-arrestin Pathway: As a consequence of phosphorylation, KOR recruits β-arrestin, activating mitogen-activated protein kinases (MAPKs) (P38, ERK (extracellular signal-regulated kinase), JNK (c-Jun N-terminal kinase)). It has been shown that this pathway is most associated with central side effects such as dysphoria, but it is also believed to participate in neuroprotection in specific cell types (e.g., microglia). **(E)** Plasma Membrane Anchoring of STAT3: In chondrocytes, activated KOR can physically interact with STAT3 at the plasma membrane, preventing its nuclear translocation and exerting a non-canonical inhibitory effect.

KOR initiates a complex network of intracellular signaling events (Figure 1) that collectively orchestrate its diverse physiological and immunological actions. A comprehensive understanding of these pathways is essential to elucidate how KOR activation translates into anti-inflammatory, cytoprotective, or neuroregulatory outcomes. The receptor can engage both canonical and non-canonical signaling cascades, with functional consequences that are highly context- and cell type–dependent.

### 2.1 Canonical inhibitory G protein (Gαi/o) pathway

KOR belongs to the class A family of G protein–coupled receptors (GPCRs) (Wu et al., 2012). Upon ligand binding, KOR couples to inhibitory Gαi/o proteins, leading to suppression of adenylate cyclase (AC) activity and a subsequent reduction in intracellular cyclic adenosine monophosphate (cAMP) levels (Taiwo and Levine, 1991). Diminished cAMP attenuates protein kinase A (PKA) activity, thereby downregulating multiple downstream signaling molecules. Since the cAMP/PKA axis functions as an upstream activator of key pro-inflammatory transcription factors—most prominently nuclear factor κB (NF-κB)—this pathway represents the classical molecular basis for KOR-mediated anti-inflammatory and analgesic effects (Che and Roth, 2021; Bruchas and Chavkin, 2010; Bohn et al., 1999). In immune cells, this suppression translates into reduced transcription of cytokines such as tumor necrosis factor α (TNF-α) and interleukin-6 (IL-6), thereby maintaining immunological homeostasis.

### 2.2 β-arrestin–dependent signaling pathway

Following receptor activation and phosphorylation, KOR recruits β-arrestin proteins that serve dual roles in receptor regulation and signal propagation (Appleyard et al., 1999). Classically, β-arrestin mediates receptor desensitization and internalization, but it also functions as a scaffold to initiate independent downstream cascades, particularly the mitogen-activated protein kinase (MAPK) family—including p38 MAPK (Bruchas et al., 2006), extracellular signal-regulated kinase (ERK) (McLennan et al., 2008), and c-Jun N-terminal kinase (JNK) (McDonald et al., 2000).

While activation of the β-arrestin pathway—specifically via p38 MAPK—drives KOR-mediated dysphoria and aversion in neurons (Bruchas et al., 2007), this signaling cascade is not inherently deleterious. As detailed in Section 3.2, the functional outcome of β-arrestin recruitment depends on the cellular context. In microglia, for instance, β-arrestin engages distinct downstream partners to confer neuroprotection (Feng et al., 2014). This dichotomy underscores that the physiological consequences of KOR signaling depend heavily on the specific proteome of the expressing cell type. Consequently, the concept of “biased agonism”—designing ligands to preferentially engage G-protein signaling while minimizing β-arrestin recruitment—remains a leading strategy to preserve therapeutic efficacy while reducing CNS-related side effects.

### 2.3 Regulation of key transcription pathways

#### 2.3.1 NF-κB pathway

Following the Gαi/o-cAMP cascade, the suppression of NF-κB acts as a central mechanism for KOR-mediated immune silencing. While largely driven by reduced PKA activity—which prevents IκB degradation and subsequent NF-κB nuclear entry—KOR modulation of inflammatory signaling can also involve PKA-independent routes. For instance, the PI3K/Akt pathway operates in parallel to suppress IκB kinase α/β-mediated NF-κB activation, thereby mitigating neuronal apoptosis and inflammation (Sun et al., 2019). The functional impact of this signaling varies by cell type: in peripheral macrophages, KOR activation potently suppresses the LPS-induced production of TNF-α, IL-1, and IL-6 (Alicea et al., 1996), whereas in CNS microglia, it downregulates human immunodeficiency virus (HIV)-1 expression and suppresses viral-mediated neuropathogenesis (Chao et al., 1996). Ultimately, this pathway provides a dual therapeutic benefit by dampening neuronal excitability to relieve pain while simultaneously resolving inflammation.

#### 2.3.2 The Janus kinase 2(JAK2)/signal transducer and activator of transcription 3(STAT3) pathway

KOR signaling also intersects with the JAK2/STAT3 axis. In certain immune and non-immune cells, KOR activation promotes phosphorylation of JAK2, which in turn activates STAT3 (Finley et al., 2011). Phosphorylated STAT3 translocates to the nucleus and induces transcription of interferon regulatory factor 2 (IRF2), a transcriptional repressor that binds to the promoter of the C-X-C chemokine receptor 4 (CXCR4) gene and downregulates its expression (Finley et al., 2011; Paun and Pitha, 2007). Through this mechanism, KOR limits chemokine signaling and immune-cell recruitment, thus contributing to its anti-inflammatory and antiviral actions.

#### 2.3.3 Non-canonical regulation of STAT3

In addition to the canonical JAK-STAT pathway, KOR regulates STAT3 through direct physical interaction. Mechanistic data indicate that activated KOR acts as a membrane anchor, sequestering STAT3 at the plasma membrane (Liu et al., 2024). This retention prevents STAT3 nuclear translocation, thereby inhibiting the transcription of pro-inflammatory cytokines and matrix-degrading enzymes (Liu et al., 2024).

These findings provide a molecular basis for the chondroprotective effects of KOR agonists reported in preclinical osteoarthritis (OA) models (Sinha et al., 2025). By preventing STAT3 from engaging its nuclear targets (Latourte et al., 2017), KOR activation limits the inflammatory cascade. This non-canonical signaling supports a dual therapeutic outcome: suppression of tissue damage alongside established analgesia.

### 2.4 PI3K/Akt/NOS cytoprotective pathway

Contrasting its inhibitory effects on inflammatory signaling, KOR activation can simultaneously engage pro-survival and cytoprotective cascades. Activation of phosphoinositide 3-kinase (PI3K) leads to phosphorylation of protein kinase B (Akt), which in turn activates endothelial and neuronal nitric-oxide synthases (eNOS/nNOS), promoting nitric oxide (NO) production (Zhang et al., 2021; Peng et al., 2009). The PI3K/Akt/NO axis mediates vasodilation, enhances microcirculatory perfusion, and suppresses apoptosis and oxidative stress (Cunha et al., 2012). In immune and endothelial contexts, this pathway contributes to the overall homeostatic and tissue-protective functions of KOR signaling, including inhibition of NOD-like receptor family pyrin domain–containing 3 inflammasome activation.

## 3 Effects of KOR on immune cells

KOR exerts complex and context-dependent regulatory effects across multiple immune cell populations. Its influence extends beyond simple anti-inflammatory actions to encompass bidirectional modulation of cellular activation, cytokine production, and chemotaxis. The overall immunological outcome of KOR engagement is shaped by several variables, including ligand structure, receptor expression level, and the tissue microenvironment.

### 3.1 Monocytes/macrophages

As central components of the innate immune system, macrophages represent the first line of defense against invading pathogens. Numerous studies have demonstrated that KOR activation suppresses pro-inflammatory signaling and cytokine release in these cells. The selective KOR agonist U50,488H dose-dependently inhibits the production of TNF-α, IL-1β, and IL-6 in both primary macrophages and macrophage-derived cell lines such as P388D1 (Alicea et al., 1996; Belkowski et al., 1995; Parkhill and Bidlack, 2006). Mechanistically, this inhibition is mediated primarily through the canonical Gαi/o–cAMP–PKA pathway, leading to suppression of NF-κB–driven transcriptional activity.

However, KOR signaling in macrophages is not uniformly inhibitory. Its effects display pronounced cell-type and ligand specificity. For instance, U50,488H fails to alter cytokine output in RAW264.7 macrophages, likely reflecting the absence or low transcriptional activity of the *Oprk1* gene in this lineage. Conversely, the naturally occurring KOR agonist salvinorin A exerts potent anti-inflammatory effects, inhibiting NO, TNF-α, interleukin-10(IL-10), and inducible nitric oxide synthase (iNOS) production in LPS-stimulated macrophages (Aviello et al., 2011; Rossi et al., 2016). This functional interaction—dependent on both KOR and cannabinoid receptor type 1 signaling—illustrates how ligand promiscuity can expand the immunomodulatory spectrum of KOR signaling.

Further evidence underscores the bidirectional nature of KOR-mediated regulation. U50,488H suppresses monocyte and neutrophil chemotaxis in primates (Miyagi et al., 2000), whereas the endogenous peptide dynorphin A promotes monocyte migration in a concentration-dependent manner (Ruff et al., 1985). Similarly, KOR activation has been reported to either inhibit or enhance phagocytic activity depending on the ligand and tissue context: U50,488H suppresses phagocytosis in murine peritoneal macrophages (Szabo et al., 1993), while dynorphin A enhances phagocytic capacity in peripheral macrophages but inhibits it in splenic macrophages (Ichinose et al., 1995; Kumar and Rai, 2011).

Such context-dependent outcomes highlight KOR’s role as a fine-tuning regulator of macrophage function rather than a purely suppressive receptor. In OA models, U50,488H attenuates M1 macrophage polarization through NF-κB inhibition (Shi et al., 2024), whereas the non-selective antagonist naltrexone exerts similar anti-inflammatory effects by targeting the Toll-like receptor 4 (TLR4)/NF-κB axis in rheumatoid arthritis (Xu et al., 2020). These findings indicate that both agonistic and antagonistic modulation of KOR-related pathways can yield anti-inflammatory outcomes, depending on disease state and ligand bias. Additionally, KOR expression itself is dynamically regulated by inflammatory mediators: interferon-γ (IFN-γ) upregulates *Oprk1* expression in J774 macrophages, suggesting the existence of a negative feedback loop to restrain excessive inflammation (Gabrilovac et al., 2012).

### 3.2 Microglia

Microglia are the resident immune cells of the CNS. Early studies demonstrated that KOR activation suppresses microglial activity, evidenced by reduced superoxide anion production (Hu et al., 1998) and inhibition of HIV-1 viral expression (Chao et al., 1996). While these protective effects were initially attributed to general anti-inflammatory signaling, subsequent research identified a cell-type specific mechanism involving β-arrestin that differs fundamentally from neuronal signaling.

In neurons, KOR recruits β-arrestin to scaffold the p38 MAPK pathway, a process that drives stress-induced aversion (Bruchas et al., 2007). In contrast, microglial β-arrestin 2 functions as an inhibitory scaffold. Upon KOR activation, it physically binds to TGF-β-activated kinase, sequestering the kinase and preventing downstream activation of the NF-κB cascade (Feng et al., 2014). This mechanistic divergence—activation of p38 in neurons versus inhibition of NF-κB in microglia—explains how KOR agonists can provide neuroprotection in inflammatory contexts while simultaneously inducing aversion through neuronal circuits.

### 3.3 T cells

T cells are the central effectors of adaptive immunity, and KOR modulates their function at multiple stages—from thymic maturation to peripheral activation. Studies in *Oprk1*-deficient mice reveal that KOR signaling is required for normal thymocyte development: knockout animals exhibit reduced thymic cellularity and a lower proportion of mature CD4^+^ T cells, despite an increased overall T-cell ratio, suggesting impaired maturation of the CD4^+^ lineage (Gavériaux-Ruff et al., 2003).

The effects of KOR on T-cell proliferation vary according to experimental context. *In vitro*, U50,488H suppresses T-cell proliferation (Guan et al., 1997), whereas *in vivo* it paradoxically promotes proliferation (Kowalski, 1998). This discrepancy highlights the importance of the *in vivo* microenvironment, where KOR signaling indirectly modulates T-cell function through effects on neurotransmitter release and antigen-presenting cell activity. Rather than being inherently stimulatory or inhibitory, KOR acts as a context-sensitive modulator that integrates neural cues with immune activation thresholds.

### 3.4 B cells

B cells mediate humoral immunity through antibody production, and KOR activation generally exerts an inhibitory influence on their function. In murine splenocyte cultures, U50,488H suppresses antibody synthesis in a concentration-dependent manner, an effect fully reversed by the selective antagonist nor-BNI (Taub et al., 1991). Correspondingly, *Oprk1* knockout mice display heightened immunoglobulin production, confirming that KOR activation negatively regulates humoral responses (Gavériaux-Ruff et al., 2003). *In vivo* studies further demonstrate that peripheral KOR activation dampens the primary antibody response, likely via direct inhibition of plasma cell differentiation (Radulović et al., 1995). Because B-cell activation depends on interactions with T helper cells and antigen-presenting cells, KOR’s regulation of humoral immunity likely involves both direct and indirect mechanisms. Future studies should aim to delineate the specific signaling events within B cells and clarify the cross-talk between KOR and co-stimulatory pathways that shape antibody responses.

### 3.5 Dendritic cells

Dendritic cells are the principal antigen-presenting cells bridging innate and adaptive immunity. Functional expression of KOR on dendritic cells has been confirmed, providing a molecular basis for their direct modulation by opioid signaling. The endogenous ligand dynorphin A suppresses T-cell proliferation by shifting the Th1/Th2 cytokine balance without affecting DC maturation or antigen-presenting capacity (Kirst et al., 2002). This selective immunomodulation allows KOR to dampen excessive T-cell activation while preserving antigen surveillance, thereby maintaining immune equilibrium.

### 3.6 Neutrophils

Neutrophils are key effectors of acute inflammation whose excessive infiltration can exacerbate tissue injury. KOR activation regulates neutrophil recruitment and function by modulating chemotaxis and adhesion. Synthetic and natural KOR agonists, including β-tetrahydropyran–salvinorin B, significantly reduce neutrophil infiltration into inflamed tissues (Paton et al., 2017). Local administration of U50,488H in temporomandibular joint inflammation models suppresses plasma extravasation and neutrophil migration in a dose-dependent manner (Chicre-Alc et al., 2012). Moreover, KOR activation indirectly limits leukocyte adhesion by downregulating intercellular adhesion molecule-1 expression on vascular endothelium, thereby improving microcirculatory perfusion during ischemia-reperfusion (I/R) injury (Lin et al., 2008). Collectively, these actions position KOR as a potential therapeutic target for neutrophil-driven inflammatory disorders.

## 4 Therapeutic applications of KOR in immune-related diseases

**FIGURE 2:**
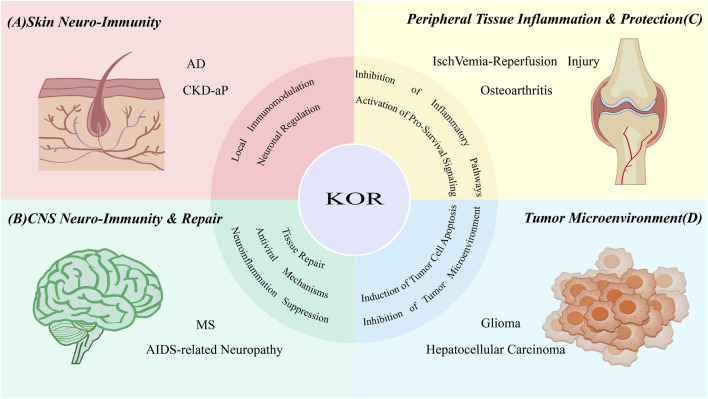
Core regulatory principles of KOR in different disease areas. The κ-opioid receptor (KOR), positioned as a central hub, exerts its function as a neuroimmune modulator through distinct core mechanisms in various pathological contexts. **(A)** Skin Neuroimmune Diseases: By directly regulating sensory neurons’ excitability and modulating local immune cells, KOR disrupts the itching-inflammation cycle. **(B)** CNS Diseases: KOR exhibits a tripartite role in repair, anti-inflammation, and antiviral activity, including promoting remyelination, inhibiting microglia and T cells, and downregulating the human immunodeficiency virus (HIV) co-receptor C-X-C chemokine receptor type 4 (CXCR4). **(C)** Peripheral Tissue Injury: KOR protects tissues by inhibiting key inflammatory pathways and activating pro-survival pathways. **(D)** Cancer: KOR exerts its anti-cancer effects by directly inducing tumor cell apoptosis and inhibiting the tumor microenvironment (e.g., angiogenesis).

KOR agonists have emerged as promising therapeutic candidates for a range of immune-mediated and neuro-inflammatory disorders. By dissecting their core mechanisms of action, it becomes evident that As illustrated in Figure 2, KOR functions not merely as an analgesic receptor but as a cross-disease regulatory hub orchestrating neuroimmune communication. In this section, we discuss representative pathological conditions where KOR-targeted modulation exerts clinically meaningful effects, highlighting its dual role in neuronal signaling and immune homeostasis.

### 4.1 Targeting the neuroimmune axis: treatment of chronic pruritus

Chronic pruritus represents a paradigmatic example of KOR’s function as a cutaneous neuroimmune regulatory hub. The pathophysiology of chronic itch involves a self-perpetuating “*itch–scratch*” cycle, wherein inflammatory mediators released by immune cells activate sensory neurons to induce pruritus, while neuropeptides secreted from these neurons further amplify local inflammation. KOR provides a unique therapeutic leverage point because of its strategic dual expression: it is present both on immune cells and keratinocytes—cells responsible for releasing pruritogens—as well as on peripheral sensory nerve endings that transmit pruritic signals (Tominaga et al., 2007). This dual localization allows KOR agonists to interrupt the neuroimmune feedback loop at two critical levels: (1) by directly reducing neuronal excitability, thereby dampening itch sensation; and (2) by indirectly attenuating immune activation within the cutaneous microenvironment. As a result, KOR-targeted therapies can achieve both rapid symptomatic relief and long-term disease modulation.

#### 4.1.1 AD

The rationale for targeting KOR in AD stems from observations of dysregulated epidermal opioid systems in patients. Clinical biopsies reveal a significant downregulation of KOR expression in the epidermis of AD lesions, suggesting that a deficiency in local KOR signaling may contribute to the intractable itch-scratch cycle (Tominaga et al., 2007).

Preclinical mechanistic studies have elucidated the dual therapeutic potential of KOR agonists in this context. In murine models of contact dermatitis, the administration of KOR agonists such as nalbuphine and nalfurafine not only suppressed scratching behavior but also exerted potent anti-inflammatory effects, characterized by the downregulation of the pruritogenic cytokine interleukin-31 (IL-31) and the upregulation of the anti-inflammatory cytokine IL-10 (Inan et al., 2019; Elliott et al., 2016). Interestingly, recent investigations utilizing difelikefalin (DFK) in a calcipotriol-induced AD mouse model suggested that its rapid antipruritic effect might be primarily mediated by peripheral neuromodulation of mechanosensitive Aβ-fibers, rather than direct immune suppression (Tamari et al., 2023). These findings indicate that KOR agonists may ameliorate AD symptoms through synergistic actions on both sensory neurons and immune cells.

Clinically, this translational potential has been validated. In a pivotal Phase 2 randomized, placebo-controlled trial (Guttman-Yassky et al., 2023), oral DFK significantly reduced itch intensity in subjects with moderate-to-severe AD-related pruritus. Notably, the drug showed pronounced efficacy in the ‘itch-dominant’ phenotype (mild skin lesions but severe itch). Furthermore, biomarker analysis from this trial confirmed that systemic KOR activation reduced the expression of key pruritus and inflammatory markers (e.g., IL-31, C-C motif ligand 17) in human skin lesions, providing clinical proof-of-concept that KOR modulation can disrupt the neuroimmune axis in AD patients (Guttman-Yassky et al., 2023).

#### 4.1.2 Chronic kidney disease-associated pruritus (CKD-aP)

The same neuroimmune regulatory principle extends to systemic disorders such as CKD-aP, a debilitating complication frequently observed in dialysis patients. Reduced KOR expression in the skin of CKD-aP patients correlates inversely with itch severity, underscoring a pathogenic role of impaired KOR signaling and establishing a clear biological indication for KOR agonist therapy (Wieczorek et al., 2020). The clinical success of DFK has provided compelling validation for peripheral KOR targeting as a viable therapeutic strategy. In a pivotal phase III randomized controlled trial, DFK demonstrated robust efficacy (specifically, significant reduction in Worst Itching Intensity scores) and safety in the management of moderate-to-severe pruritus among hemodialysis patients (Fishbane et al., 2020). This achievement culminated in its approval by the U.S. Food and Drug Administration in 2021, marking the first global approval of a peripherally restricted KOR agonist.

DFK’s therapeutic advantage stems from its rational molecular design. As a structurally modified peptide, DFK possesses physicochemical properties that prevent blood–brain barrier penetration, thereby confining its activity to the peripheral nervous and immune systems. This spatial restriction enables potent anti-pruritic and anti-inflammatory actions at the peripheral level while minimizing CNS-mediated adverse effects (Tey and Yosipovitch, 2011). Beyond CKD-aP, DFK’s clinical success establishes a broader proof of concept: precise pharmacological modulation of the peripheral neuroimmune axis can yield safe, effective therapies for a variety of pruritic and inflammatory disorders. Its mechanism—concurrently attenuating peripheral neuronal excitability and immune activation—represents a model for next-generation neuroimmune–targeted therapeutics.

### 4.2 Modulating CNS inflammation and promoting repair: neurological diseases

Within the CNS, KOR serves as a pivotal neuroimmune regulator capable of modulating multiple pathological processes. Beyond suppressing microglia-driven innate immune responses and limiting adaptive immune cell infiltration, KOR activation confers direct neuroprotective effects and uniquely facilitates remyelination. These multifaceted actions position KOR as a central therapeutic node in neuroinflammatory and demyelinating disorders.

#### 4.2.1 MS

MS is a chronic autoimmune demyelinating disorder characterized by aberrant infiltration of immune cells into the CNS, resulting in inflammation, axonal damage, and myelin loss. Although conventional therapies—such as immunomodulators and immunosuppressants—can attenuate immune-mediated injury, they largely fail to promote myelin repair or restore neurological function. Consequently, KOR agonists offer a distinct therapeutic advantage by simultaneously suppressing neuroinflammation and enhancing endogenous myelin regeneration.

##### 4.2.1.1 Direct pro-myelinating and reparative effects

Preclinical studies demonstrate that KOR activation promotes oligodendrocyte precursor cell (OPC) differentiation and remyelination, leading to functional recovery in experimental MS models (Du et al., 2016; Tangherlini et al., 2019; Thell et al., 2016). Pharmacological activation of KOR by agonists such as U50,488H, nalfurafine, and the semi-synthetic Salvinorin A derivative ethoxymethyl ether Salvinorin B (Paton et al., 2021) markedly enhances the maturation of OPCs. Crucially, Mei et al. extended these findings to a human context, demonstrating that KOR activation also promotes the differentiation of human induced pluripotent stem cell-derived OPCs *in vitro* (Mei et al., 2016). These effects are abolished in *Oprk1*-deficient mice, confirming that the remyelination process is KOR-dependent (Du et al., 2016; Paton et al., 2021; Mei et al., 2016).

##### 4.2.1.2 Immunomodulatory and anti-inflammatory actions

In addition to promoting myelin repair, KOR agonists exert potent immunomodulatory effects. Agents such as nalfurafine (Denny et al., 2021) and the orally active cyclotide [T20K] kalata B1 (Thell et al., 2016) reduce the infiltration of peripheral immune cells into the CNS. Specifically, novel quinoxaline-based agonists have been shown to delay experimental autoimmune encephalomyelitis onset by blocking effector T cell activation (Tangherlini et al., 2019; Denny et al., 2021). Moreover, KOR activation mitigates neuroinflammation by inhibiting microglial activation, thereby reducing the local production of cytotoxic mediators and inflammatory cytokines.

Collectively, these findings establish KOR as a dual-function therapeutic target in MS—capable of restraining neuroinflammatory cascades while concurrently activating intrinsic repair pathways. Such pleiotropic effects distinguish KOR agonists from traditional immunosuppressive agents and position them as promising candidates for the next generation of remyelination-promoting therapies. However, it must be noted that these findings are currently limited to rodent models (e.g., experimental autoimmune encephalomyelitis, cuprizone) and *in vitro* human cell assays. Clinical trials are yet to confirm whether these neuro-reparative effects can be translated to MS patients.

#### 4.2.2 Acquired immunodeficiency syndrome (AIDS)-associated neuropathy

AIDS results from HIV infection, leading to immune collapse and neurological complications. *In vitro* studies utilizing human primary cells have provided compelling evidence that KOR agonists confer neuroprotective and antiviral benefits through multiple complementary mechanisms.

##### 4.2.2.1 Inhibition of viral replication and neurotoxicity

KOR activation suppresses HIV-1 replication in major host cell types, including macrophages (Chao et al., 2001), microglia (Chao et al., 1996), and CD4^+^ T cells (Peterson et al., 2001). Beyond direct antiviral effects, KOR signaling attenuates the release of neurotoxic factors, such as quinolinic acid, from infected microglia, thereby protecting neurons from excitotoxic injury (Chao et al., 2000).

Furthermore, KOR ligands have been shown to block the potentiation of HIV-1 expression induced by substances of abuse like cocaine (Gekker et al., 2004).

##### 4.2.2.2 Regulation of inflammatory chemokine signaling

In human astrocytes, KOR agonists downregulate astrocytic production of the chemokine C-C motif ligand 2, which is critical for recruiting inflammatory monocytes to the CNS (Sheng et al., 2003). Moreover, KOR activation decreases cellular susceptibility to HIV infection by suppressing expression of CXCR4, a principal co-receptor for viral entry (Finley et al., 2011).

These *in vitro* findings suggest that KOR agonists possess a unique multi-target profile against HIV-associated neuropathology. However, it is important to note that these mechanisms have yet to be validated in clinical trials. Future translational research is needed to determine whether these cellular effects can translate into improved neurological outcomes in HIV patients.

### 4.3 Regulation of peripheral inflammation and tissue protection

Beyond its central effects, KOR exerts pivotal immunomodulatory and cytoprotective functions across multiple peripheral organ systems. By attenuating inflammatory cascades, modulating immune cell polarization, and activating pro-survival signaling pathways, KOR contributes to the preservation of tissue integrity and systemic homeostasis.

#### 4.3.1 Arthritis

The therapeutic rationale for targeting KOR in OA is supported by clinical findings that KOR expression is significantly downregulated in the synovium of OA patients. This suggests that a loss of endogenous protective signaling contributes to disease pathogenesis (Shi et al., 2024). Preclinical studies indicate that KOR agonists protect the joint through two distinct mechanisms.

First, within the synovium, KOR activation restrains macrophage polarization toward a pro-inflammatory M1 phenotype, an effect mediated by suppression of the NF-κB signaling axis (Shi et al., 2024). Concurrently, KOR acts directly on chondrocytes to inhibit cartilage catabolism via the STAT3 pathway (Latourte et al., 2017). More recent work suggests a novel mechanism whereby activated KOR sequesters STAT3 at the plasma membrane, preventing its nuclear translocation and subsequent transcriptional activity (Liu et al., 2024).

In animal models of OA, the combined inhibition of both NF-κB-driven synovitis and STAT3-mediated chondrocyte degradation preserves joint architecture and reduces subchondral bone pathology (Sinha et al., 2025). It is important to note, however, that these promising findings are currently limited to preclinical models, and the translation of these chondroprotective and anti-inflammatory effects into clinical OA treatment requires further investigation.

#### 4.3.2 Gastrointestinal disorders

KOR signaling also plays a vital role in maintaining gastrointestinal immune balance. In various experimental models of intestinal inflammation, KOR agonists ameliorate mucosal injury and dysmotility through both neural and immune mechanisms. For instance, the naturally derived agonist salvinorin A markedly alleviates colitis by engaging both KOR and cannabinoid receptor type 1, thereby reducing mucosal inflammation and restoring epithelial integrity (Ranganathan et al., 2012). Similarly, in murine models of allergic diarrhea, activation of KOR by U50,488H suppresses both intestinal mast cell infiltration and Th2-type immune responses, leading to significant attenuation of symptoms (Duncker et al., 2012). Emerging evidence also suggests that KOR contributes to the regulation of visceral hypersensitivity, a core pathological feature of irritable bowel syndrome (IBS) (Chey et al., 2015); however, clinical data on KOR-selective agonists in IBS remain limited. Nevertheless, peripherally restricted KOR agonists such as asimadoline have been investigated in clinical trials for IBS, showing promise in managing visceral pain and bowel symptoms, particularly in diarrhea-predominant IBS, without CNS liabilities (Mangel et al., 2008). These findings collectively indicate that KOR functions as a bidirectional regulator of gut–immune homeostasis, capable of modulating enteric neuronal signaling and immune cell activation to alleviate intestinal inflammation.

#### 4.3.3 I/R injury

In models of I/R injury affecting vital organs such as the heart, kidney, and brain, KOR activation confers robust tissue-protective and anti-inflammatory effects. The protective mechanisms involve multiple convergent pathways: Inhibition of pro-inflammatory signaling: KOR agonists suppress the TLR4/NF-κB cascade, leading to decreased TNF-α release and reduced neutrophil infiltration (Lin et al., 2008; Lin et al., 2013). Activation of cytoprotective signaling: Through engagement of the PI3K/Akt axis, KOR stimulation enhances cellular survival, limits apoptosis, and improves microcirculatory perfusion (Sun et al., 2019; Liu et al., 2018; Wu et al., 2011). Cross-talk with JAK2/STAT3 and HIF-1α pathways: In cardiopulmonary bypass models, KOR activation mitigates postoperative neuroinflammation and cognitive dysfunction via inhibition of the JAK2/STAT3 pathway, while also protecting the intestinal barrier by suppressing oxidative stress through the NF-κB/HIF-1α axis (Zhang et al., 2020). Through these coordinated actions, KOR functions as an endogenous cytoprotective modulator that bridges anti-inflammatory and pro-survival pathways. Its ability to regulate both immune and metabolic components of tissue injury underscores its therapeutic potential in reperfusion-related syndromes.

#### 4.3.4 Hypoxic pulmonary hypertension

KOR signaling has also been implicated in the regulation of pulmonary vascular remodeling under hypoxic stress. KOR represents the predominant opioid receptor subtype expressed in pulmonary vascular tissues and is upregulated during early hypoxia, where its activation induces vasodilation through the PI3K/Akt/eNOS pathway (Peng et al., 2009).

Mechanistically, KOR activation has been shown to counteract the pro-hypertensive effects of the calcium-sensing receptor by inhibiting its downstream MAPK signaling cascade, thereby preventing pulmonary artery smooth muscle cell proliferation and vasoconstriction (Li et al., 2020). However, as hypoxia persists, the inflammatory cytokine IL-6 released from macrophages suppresses KOR expression via the STAT3/miR-153-3p signaling axis, establishing a self-amplifying inflammatory loop that accelerates vascular remodeling and disease progression. Pharmacological activation of KOR interrupts this pathological feedback circuit, restoring receptor expression and endothelial homeostasis. Additionally, KOR agonists inhibit neutrophil and T-helper cell activation. Collectively, these preclinical findings position KOR as a vasoprotective and anti-remodeling mediator in hypoxic pulmonary hypertension, functioning to decouple inflammatory and proliferative signaling within the pulmonary vasculature.

## 5 KOR and anti-tumor effects

The relationship between opioid signaling and cancer progression has long been a subject of intense debate. Early studies suggested that opioids might facilitate tumor growth and metastasis; however, this oversimplified view has been increasingly refuted by accumulating mechanistic and clinical evidence. It is now recognized that the biological effects of opioids are highly context-dependent (Yuval et al., 2022). Crucially, the specific opioid agent administered plays a determinative role. As demonstrated by classic structure-activity studies, the immunosuppressive potential of opioids is not correlated with their analgesic potency but is strictly dictated by their chemical structure (e.g., substitutions at the C6 position (Sacerdote et al., 1997). For instance, while morphine (a phenanthrene) activates TLR4 to drive inflammation and potential metastasis (Hutchinson et al., 2010), synthetic phenylpiperidines like fentanyl lack this off-target activity and exhibit a distinct, often neutral, immunomodulatory profile. Beyond the specific agent used, the specific opioid receptor subtype engaged is of paramount importance. For example, while pro-tumorigenic effects have been reported in certain subtypes of clear cell renal cell carcinoma (Silagy et al., 2020) and non-small cell lung cancer (Connolly et al., 2021a; Connolly et al., 2021b), opposite outcomes—characterized by anti-proliferative and pro-apoptotic effects—have been observed in triple-negative breast cancer and colon adenocarcinoma. This dichotomy underscores the need to move beyond generalized discussions of “opioids” toward a receptor subtype–specific understanding of their roles in tumor biology.

### 5.1 KOR as a tumor-suppressive regulator

Among the opioid receptor subtypes, KOR has emerged as a promising target with predominantly tumor-suppressive properties. A growing body of evidence indicates that KOR expression correlates inversely with tumor aggressiveness and patient mortality across several solid malignancies. In gliomas, high KOR expression is associated with lower tumor grade and improved overall survival (Lambert and Mincer, 2025). Similarly, in hepatocellular carcinoma (Chen et al., 2017) and breast cancer (Shi et al., 2023), KOR levels are significantly reduced in tumor tissues relative to adjacent normal tissues, and diminished expression correlates with poor prognosis and higher recurrence risk. These observations suggest that physiological KOR activity may contribute to maintaining tissue homeostasis and restraining tumorigenesis.

Mechanistic studies further demonstrate that KOR activation exerts direct anti-tumor effects through multiple intracellular pathways. Pharmacological activation of KOR has been shown to inhibit tumor cell proliferation and induce apoptosis via modulation of the p38 MAPK cascade (Lambert and Mincer, 2025; Diao et al., 2000). In estrogen receptor–positive breast cancer cells, KOR agonists suppress cell growth and survival signaling (Shi et al., 2023) while in melanoma models, compounds such as U50,488H and nalfurafine inhibit tumor angiogenesis and growth (Zhou Q. et al., 2022). Importantly, single-cell transcriptomic analyses have revealed that in certain malignancies, KOR constitutes the predominant—or even exclusive—opioid receptor subtype expressed within the tumor microenvironment (Montagna et al., 2021), providing a compelling rationale for its exploitation as a precision oncologic target.

### 5.2 Context-dependent and bidirectional effects

Despite these generally protective findings, KOR’s role in cancer is not universally suppressive. Isolated studies have reported that KOR overexpression can enhance invasiveness in specific breast cancer cell lines and may be associated with poorer outcomes in selected subtypes of liver cancer and non-small cell lung cancer (Chen et al., 2017; Tripolt et al., 2021). Such apparent discrepancies likely reflect context-specific signaling biases determined by tumor origin, receptor density, ligand structure, and the metabolic and immunological microenvironment.

Rather than negating KOR’s therapeutic potential, these contrasting observations highlight its situational dependence—a hallmark of GPCR signaling in heterogeneous disease contexts. The biological outcome of KOR activation is therefore shaped not only by receptor engagement but also by the dynamic interplay between oncogenic signaling networks and local immune or stromal cues.

### 5.3 Therapeutic and translational implications

Overall, the preponderance of experimental and clinical evidence supports a predominantly tumor-suppressive function for KOR in solid malignancies. This understanding provides a strong mechanistic foundation for the development of selective KOR agonists as potential anti-cancer agents. Beyond their established roles in analgesia, such compounds may exert dual therapeutic benefits—offering effective pain relief while synergizing with existing cancer treatments such as chemotherapy and immune checkpoint blockade.

This conceptual shift—from opioids as symptomatic analgesics to neuroimmune modulators with anti-tumor potential—opens new avenues for precision oncology. Selectively targeting KOR could enable the design of “analgesic–anti-tumor hybrids” that preserve central pain control while actively contributing to the suppression of tumor progression and metastasis.

## 6 Challenges and future perspectives

Despite compelling preclinical evidence supporting the immunomodulatory and tissue-protective potential of KOR–based therapies, the translation of these findings into clinical success remains challenging. Several key issues must be addressed to bridge the gap between mechanistic insight and therapeutic application. Future research should focus on the following strategic directions.

### 6.1 Rational design and development of next-generation ligands

The main challenge in KOR drug development is to separate therapeutic effects from CNS-mediated side effects. Two main strategies have been pursued: peripheral restriction and biased agonism. The first is exemplified by DFK, a peripherally acting agonist approved for pruritus, which avoids central side effects by not crossing the blood-brain barrier (Fishbane et al., 2020).

The second strategy, biased agonism, aims to selectively activate therapeutic G-protein pathways over β-arrestin pathways linked to adverse effects. However, the clinical relevance of this model remains debated. For example, nalfurafine, a clinically used KOR agonist, shows a variable bias profile depending on the assay system used (Zhou Y. et al., 2022; El Daibani et al., 2023). This inconsistency makes it difficult to use *in vitro* bias as a sole predictor of clinical safety. These findings suggest that a favorable safety profile may also depend on other factors. For instance, a drug’s specific pharmacokinetic properties, or its status as a partial agonist (low intrinsic efficacy), might be just as important as its signaling bias in creating a therapeutic window.

### 6.2 Deciphering the situational dependence of KOR signaling

As highlighted throughout this review, KOR signaling exhibits bidirectional and context-dependent effects, with agonists and antagonists occasionally producing overlapping outcomes in specific disease models, and receptor activation displaying both pro- and anti-tumoral functions across cancer subtypes. Elucidating this situational dependence represents a major conceptual and experimental Frontier. A systematic understanding of how the disease microenvironment—including cytokine composition, local hypoxia, oxidative stress, and pH—modulates KOR conformation, receptor–effector coupling, and downstream transcriptional responses is urgently needed. Integrating single-cell and spatial transcriptomics, ligand-bias profiling, and metabolomic mapping will help delineate how microenvironmental cues reshape KOR’s signaling topology. Such knowledge is indispensable for patient stratification, mechanistic biomarker discovery, and predicting therapeutic responsiveness to KOR-targeted interventions. Finally, sexual dimorphism is a critical factor influencing KOR signaling that cannot be ignored. Previous pharmacological studies have shown that KOR-mediated analgesia differs significantly between sexes; for instance, KOR agonists are often more potent in male rodents compared to females (Craft, 2003). This difference is partly linked to sex hormones, such as estrogen, which can modulate receptor expression and signaling efficiency (Rasakham and Liu-Chen, 2011). Considering that many neuroimmune disorders, including MS and autoimmune arthritis, are more prevalent in females (Klein and Flanagan, 2016), it is likely that the immunomodulatory effects of KOR also vary by sex. Therefore, future translational research must clarify whether KOR ligands exert the same neuroimmune benefits in both sexes, rather than assuming uniform efficacy.

### 6.3 Combination strategies and biomarker development

Given its multifaceted role at the neuroimmune interface, KOR represents an ideal candidate for combination therapies. In autoimmune and inflammatory diseases, KOR agonists could act synergistically with disease-modifying antirheumatic drugs or biologics to enhance efficacy while reducing required doses. In oncology, modulating the tumor immune microenvironment via KOR signaling may sensitize tumors to immune checkpoint blockade or cytotoxic therapies.

Parallel to these pharmacological strategies, biomarker discovery is essential to guide precision treatment. Quantifying KOR expression or activity in immune cell subsets may serve as a predictive indicator of disease severity and therapeutic response in conditions such as chronic kidney disease–associated pruritus or inflammatory arthritis. Development of noninvasive imaging tracers and transcriptomic biomarkers will further enable dynamic monitoring of KOR activity *in vivo*, refining clinical decision-making and patient selection.

### 6.4 Proposed clinical framework: a stratified approach

Synthesizing the pharmacological and pathological evidence presented above, we propose a stratified framework for clinical translation based on the specific neuroimmune requirements of each disease state:

1. The Peripheral Axis (OA):

For OA, the therapeutic rationale parallels pruritus. Since OA pain is largely driven by peripheral nociceptor sensitization and synovial inflammation, peripherally restricted KOR agonists offer a strategic advantage. By confining drug action to the joint microenvironment, it is possible to leverage the receptor’s anti-inflammatory and analgesic effects while avoiding blood-brain barrier penetration that leads to central adverse effects.

2. The Central Axis (MS):

In contrast, CNS disorders like MS require a fundamentally different pharmacological approach. Because the therapeutic targets-remyelination and microglial modulation-lie within the CNS, blood–brain barrier penetration is essential. Therefore, drug development for this axis must focus on G-protein-based agonists. These ligands are designed to activate the pro-reparative G-protein pathways required for oligodendrocyte differentiation, while minimizing the β-arrestin recruitment that mediates dysphoria and sedation.

3. The Oncologic Axis (Cancer Pain):

In the context of Cancer Pain, KOR agonists present a mechanistic alternative to traditional MOR. Unlike morphine, which compromises immune surveillance via off-target TLR4 activation, KOR ligands lack this specific immunosuppressive liability. Furthermore, for malignancies that retain high KOR expression, these agents may offer a dual benefit: providing analgesia through neuronal inhibition while potentially restraining tumor cell proliferation through the mechanisms discussed in Section 5.

### 6.5 Limitations of this review

Several limitations should be considered. First, this is a narrative review, not a systematic one. This means the literature was not selected using a rigid protocol like PRISMA, and we did not formally assess the quality of evidence for each study. While our aim was to synthesize a conceptual framework, this approach may have introduced selection bias.

Second, many of the mechanistic insights discussed are derived from preclinical studies in animal or cell-based models. The translation of these findings to human clinical settings requires further validation, and this remains a critical gap in many of the areas covered.

Third, because this review covers a broad range of topics from molecular signaling to multiple diseases, the discussion for any single area is concise and should not be considered exhaustive.

Finally, our literature search was primarily conducted using the PubMed database. Consequently, relevant articles indexed exclusively in other databases may have been missed.

## 7 Conclusion

KOR stands at the crossroads of neurobiology and immunology, acting as a pivotal regulator of the neuroimmune dialogue. Through the modulation of core signaling axes—including NF-κB, PI3K/Akt, and STAT3—KOR orchestrates diverse biological processes ranging from inflammation resolution and tissue repair to tumor suppression. The successful clinical translation of the peripherally selective agonist DFK provides proof-of-concept that rationally engineered KOR ligands can achieve therapeutic efficacy while minimizing CNS adverse effects. Nonetheless, the intrinsic complexity and situational plasticity of KOR signaling continue to pose formidable challenges. Future efforts must therefore prioritize the design of pathway-selective agonists, the mapping of microenvironment-dependent responses, and the integration of biomarker-guided combination therapies.

In conclusion, targeted modulation of KOR offers a transformative therapeutic paradigm for diseases characterized by neuroimmune dysregulation. By coupling mechanistic precision with translational innovation, next-generation KOR-based interventions hold the promise of reshaping clinical strategies across immunology, neurology, and oncology.

## 8 Literature search strategy

The literature for this review was identified through a primary search of the PubMed database, initially completed in March 2025. This search was supplemented by continuous literature monitoring throughout the manuscript’s preparation to incorporate the latest findings.

Core search terms included “kappa-opioid receptor”, “κ-opioid receptor”, “KOR”, and “*Oprk1*”. These were combined with secondary keywords such as “neuro-immunomodulation”, “inflammation”, “biased agonism”, “microglia”, “immun*” (to capture related terms), and specific disease states like “atopic dermatitis” and “multiple sclerosis”. Articles were selected for their relevance to the molecular mechanisms or therapeutic potential of KOR in neuroimmune contexts. Primary research articles and reviews were considered, and their reference lists were manually screened to identify additional publications.

## Glossary

KOR: κ-opioid receptor
CNS: central nervous system
MOR: μ-opioid receptor
Gαi/o: inhibitory G protein
GPCRs: G protein–coupled receptors
AC: adenylate cyclase
cAMP: cyclic adenosine monophosphate
PKA: protein kinase A
NF-κB: nuclear factor κB
TNF-α: tumor necrosis factor α
IL-6: interleukin-6
MAPK: mitogen-activated protein kinase
ERK: extracellular signal-regulated kinase
JNK: c-Jun N-terminal kinase
JAK2: Janus kinase 2
STAT3: Signal Transducer and Activator of Transcription 3
IRF2: Interferon Regulatory Factor 2
CXCR4: C-X-C chemokine receptor type 4
OA: osteoarthritis
PI3K/Akt: Phosphoinositide 3-kinase/Protein Kinase B
NOS: Nitric Oxide Synthase
NO: nitric oxide
IFN-γ: interferon-γ
NSCLC: non-small cell lung cancer
AD: Atopic Dermatitis
Il-31: interleukin-31
Il-10: interleukin-10
DFK: Difelikefalin
CKD-aP: Chronic Kidney Disease-Associated Pruritus
MS: Multiple Sclerosis
AIDS: Acquired Immunodeficiency Syndrome
HIV: human immunodeficiency virus
IBS: irritable bowel syndrome
I/R: Ischemia-Reperfusion
TLR4: Toll-like receptor 4
